# Perspective of Indian parents on physiotherapy rehabilitation in pediatric oncology: a qualitative phenomenological study protocol

**DOI:** 10.3389/fonc.2026.1870939

**Published:** 2026-09-30

**Authors:** Dhanush Kotian, Asir John Samuel

**Affiliations:** Yenepoya Physiotherapy College, Yenepoya (Deemed to be University), Naringana, Mangaluru, Karnataka, India

**Keywords:** family centered care, India, lived experience, parents perspectives, pediatric oncology, physiotherapy rehabilitation, qualitative research

## Abstract

**Background:**

Advances in pediatric oncology treatment have had a positive impact on the childhood cancer survival rates across the world. Despite this, children who are receiving treatment can experience treatment related physical impairments such as fatigue, muscle weakness, less mobility, and various day to day functional limitations, and this can quietly harm their quality of life. In this context, the need for physical rehabilitation increases as it supports physical functioning and generally supports well-being throughout the duration of treatment. Despite the increase in evidence showing that rehabilitation matters in pediatric oncology, the use of physiotherapy services stays uneven, especially in low- and middle-income countries, including India. Parents, who act as the main caregivers as key healthcare decision makers, strongly shape whether and how their child can take part in rehabilitation. However, the evidence on how Indian parents experience and make sense of physiotherapy rehabilitation during their child’s cancer journey is limited. Therefore, this study is designed to explore and understand the lived experiences and meaning-making of Indian parents regarding physiotherapy rehabilitation during their child’s cancer treatment.

**Methods:**

A phenomenological qualitative design will be employed in this study to investigate the lived experiences of Indian parents of children with cancer who are receiving physiotherapy rehabilitation. The participants who have a child aged 8-17years diagnosed with cancer and exposed to physiotherapy will be parents or primary caregiver of children will be recruited from a pediatric oncology center at a tertiary care hospital by using a purposive sampling method. Data will be collected from 8-17years old child’s parents or primary caregiver of children diagnosed with cancer and exposed to physiotherapy until data and thematic saturation is achieved. Data collection will be carried out through semi-structured in-depth interviews in English or Kannada using an interview schedule. The interview schedule will be validated by experts using the Content Validity Index and the Delphi technique. The interviews will be audio recorded, transcribed and translated in the language used for the interview and analyzed using Braun and Clarke’s approach of reflexive thematic analysis. Trustworthiness, credibility, confirmability, dependability, and transferability will be achieved through bracketing, reflexivity, independent coding, and audit trail.

**Discussion:**

This phenomenological study will offer valuable insights into the meaning of physiotherapy rehabilitation to Indian parents in the context of pediatric oncology and how it is experienced. An understanding of parental beliefs, expectations, emotions and barriers to physiotherapy rehabilitation will potentially help guide culturally appropriate, family-centered rehabilitation interventions and facilitate the integration of physiotherapy into pediatric oncology care pathways in low- and middle-income settings.

## 1 Introduction

Childhood cancer remains an important health issue facing children and adolescents around the world. Developments in the multimodal therapy of childhood cancer have significantly increased the survival rates of affected individuals, and thus there is an expanding number of childhood cancer survivors. (1–4). Even with increased survival, cancer and the treatment can lead to diverse physical effects and functional impairments, such as fatigue, decreased muscle strength and mobility, cardiopulmonary issues, weakened bones and poor quality of life. (2, 5, 6).

Organized physical activity and exercise interventions are becoming recognized as key components in the management of children and adolescents with cancer. Physical activity and exercise may provide benefits, including improved cardiopulmonary function, muscle strength and endurance, mobility, and participation in life skills. (7–10) But, the proportion of children and childhood cancer survivors engaging in optimal level of physical activity are insufficient. The factors such as treatment side effects, longer duration of hospital stay, persistent fatigues, fear of the injury and accidents, social disorganization and lack of skilled supervision services may lead them to do lesser physical exercise and rehabilitative exercise (7, 8, 11).

Rehabilitation in pediatric oncology is a multidisciplinary approach which attempts to minimise treatment side effects while optimizing the child’s participation in both their everyday activities and in the treatment process itself. Physiotherapy is a central part of this rehabilitation and address those movement related difficulties through therapeutic exercise, locomotion activities, breathing exercises, fatigue control and rehabilitation programs (9, 10, 12, 13).

Parent participation is especially important in pediatric rehabilitation since parents provide significant care to the child and participate in medical decision-making across the course of cancer treatment. Parents of children with cancer may suffer emotional strain, caregiving burden, uncertainty about treatment outcomes, and difficulty balancing caregiving and family demands (14, 15). In family-centered care, it is understood that parents can contribute meaningfully and positively to treatment planning, as well as to communication with healthcare providers, in sharing knowledge and information in decision-making, to provision of care, to rehabilitation and health education of caregivers may affect health professionals’ confidence as caregiver, which has potential impact on experience of rehabilitation. These include parental fears related to the child’s safe, ignorance of benefits of physical rehabilitation, the prioritization of medical care above supportive services could affect the participation of the child in physical activity and physical therapy (11, 16–18).

There is some evidence that parents’ attitudes and experiences can affect how children take part in physical activity and rehabilitation. Parental perceptions, concerns, barriers and facilitators have been documented in high-income countries which could affect how children with cancer take part in rehabilitation and physical activity (8, 11, 16–19). These findings suggest the need to consider both parents’ roles not only as providers of care for the child but also in their perception and experience throughout the child’s rehabilitative path.

However, the evidence base may not be generalisable from high- income contexts to low- and middle-income settings as a result of disparities in health service systems, traditions, resources, and modes of delivery. In India, a range of factors may impede the uptake and retention of rehabilitation services: Lack of awareness about rehabilitation services, lack of funds, problems with traveling, limited health-care resources, primacy of acute medical needs, and restricted access to pediatric rehabilitation specialty services. (20) Despite increasing childhood cancer survival in India (21), The extent to which physiotherapy is part of pediatric oncology pathways varies significantly. While there has been some research done in India focusing on medical outcomes and psychosocial issues; and palliative care and end-of-life care; relatively less research has focused on parental experience of rehabilitation (22–24). Therefore, the need for the qualitative evidence regarding Indian parent’s experience with regard to physiotherapy rehabilitation to their child’s cancer care is contextually needed.

Phenomenology is a methodological approach suitable for inquiry of personally valued, affect-related, context-bound phenomena that are mediated and interpreted by meanings ascribed to experience by participants. Phenomenological research attempts to discover how individuals experience a phenomenon, how individuals interpret that experience and to how participants assign meaning to that experience in their specific situation (25–27). The experience of parents in pediatric oncology rehabilitation is multifaceted and can be shaped by: the nature of their child’s disease; the cancer trajectory; care-giving demands; communication with healthcare providers and their sociocultural context. Thus, parental experience in the context of pediatric oncology rehabilitation cannot be understood without considering how parents view and respond to physiotherapy beyond their thoughts or beliefs about it, in other words how they actually experience, interpret and give meaning to rehabilitation through their child’s journey of suffering with cancer.

While there is quantitative data that has shown promising results regarding physical activity and rehabilitation in pediatric oncology, few qualitative data have explored the perceptions and meaning making of Indian parents in experiencing physiotherapy rehabilitation. Understanding these lived experiences can further help in developing family-centered culturally specific rehabilitation programs. Hence, this study seeks to describe and understand the lived experiences and meaning making of Indian parents of a child with cancer with respect to physiotherapy rehabilitation.

## 2 Materials and methods

### 2.1 Study design

The research will use a phenomenological qualitative design to study parental lived experiences and their understanding of physiotherapy rehabilitation. The research method of phenomenological study used here requires researchers to explore the essential nature of a phenomenon by studying how people experience it in their everyday life. The current research focus for this study was the experience of parents of a child receiving physiotherapy rehabilitation for cancer and so the research aims to determine what this experience means and how it is interpreted by the parents. (25, 28). In this research, phenomenology is selected as appropriate, since parents’ experiences of physiotherapy rehabilitation during their child’s cancer therapy can be seen as personal, emotional, social and cultural. The current research study uses a phenomenological research method (29) to investigate how Indian parents view and understand physiotherapy rehabilitation during their child’s cancer treatment process. During the research process, the investigators will conduct reflexivity and bracketing (epoché) by reflecting on and attempting to diminish their own pre-existing assumptions, clinical experience and philosophical perspectives on physiotherapy rehabilitation and pediatric oncology practices prior to and during data collection and analysis. Reflexivity comments will be recorded during data collection and analysis in order to increase methodological transparency and phenomenological rigor.

The study will take place at the Pediatric Oncology Unit located in the tertiary care teaching hospital in India. The process of recruiting participants is still active because it has not reached completion at the point when the manuscript was submitted. Interviews will be carried out by trained researchers who are not directly involved in the child’s care, so as to reduce the potential for power imbalance and response bias.

### 2.2 Theoretical framework

The research uses a phenomenological framework that enables researchers to study how people experience life and assign meaning to their life experiences. Phenomenology is concerned with how individuals perceive the world and assign meaning to their existence through lived experience. Applying a phenomenological perspective to pediatric oncology rehabilitation emphasizes an understanding of how parents perceive their experiences of physiotherapy rehabilitation, how parents respond emotionally to the experience of their child’s treatment and how parents interpret rehabilitation in the context of their sociocultural position and parenting roles. The research uses phenomenology to study parents’ understanding of their child’s physiotherapy rehabilitation process during cancer treatment. In Family-Centered care, parents are seen as partners of the child’s treatment and rehabilitation. Their beliefs, emotions, caregiving responsibilities and relationships with care providers could influence their involvement in the rehabilitation process and continuation of care.

The research uses a family-centered care perspective which considers parents to be the main caregivers and important decision-makers in Pediatric oncology treatment. The perspective shows how parental beliefs and values and real-life experiences determine their healthcare decisions and which therapies they will use to support their recovery process including physiotherapy rehabilitation. Parents need to understand this framework because it helps them recognize the factors that help and hinder their rehabilitation service experience (11, 16–18). The research study uses two frameworks to direct its design, data gathering and data analysis process because they focus on understanding how participants experience their lives and their roles as caregivers who make medical decisions. By combining the two philosophies, a conceptual structure can be devised to examine how parents’ experiences and perceptions of physiotherapy rehabilitation may be shaped within their role as care giver and health care decision makers throughout childhood cancer.

Phenomenology provides a methodological and philosophical basis from which to address parents’ experienced-worlds and meaning making. A family-centered- care focus can provide the broader context within which to explore the position of parents as care-givers and decision-makers in pediatric oncology rehabilitation.

### 2.3 Ethical consideration

The qualitative study protocol has been approved by the scientific review board of Yenepoya Physiotherapy College (SRB/Faculty/93/2025) on 17/12/2025 and received ethical approval from Yenepoya Ethics Committee-3 of Yenepoya (Deemed to be University), Deralakatte, Karnataka, India with a unique protocol reference number YEC-3/2026/005 on 14/02/2026 for the duration of the study from 14/02/2026 to 13/02/2027. The ethics committee granted study approval for its defined time period, and all extra time needs, will be submitted to the ethics committee for yearly extensions. The study protocol was registered by the researchers in the open-access platform https://ctri.nic.in/ on 24-02-2026 (CTRI/2026/02/104603).

The study will follow the ethical standards established in the Declaration of Helsinki (30) and the National Ethical Guidelines for Biomedical and Health Research involving Human Participants created by the Indian Council of Medical Research (31). The complete information about the objectives, procedure and confidentiality measures of the study will be explained to all the respondents and their right to withdraw from the study will be communicated. Informed consent will be obtained from all the participants before their entry to the study, including consent for the audio-recorded interviews.

### 2.4 Recruitment

The research team will select participants through purposive sampling from the Pediatric Oncology Unit of a tertiary care hospital. Potential participants will first be screened using information from the treating clinical team. Approaching potential participants will be conducted by a member of the research team who is not involved in the child’s clinical care to avoid the risk of coercion. Parents or primary caregivers of children aged 8 to 17 years who are currently undergoing or have completed physiotherapy rehabilitation for their oncology treatment will be eligible to participate in the study if they can speak English and/or Kannada. Participants will be included only if their child has received physiotherapy rehabilitation services and is able to provide experiences related to such care, either during inpatient or outpatient treatment. The study is open to both mothers and fathers. However, only one parent/primary caregiver per child will be included in the study to prevent duplicate experiences. In this study, the primary caregiver is defined as the mother or father or legal guardian with major responsibility for the child’s daily care and decisions concerning treatment. Parents/primary caregivers of children (8–17 y) with cancer, including leukemia, lymphoma, brain tumors, bone tumors, and solid organ tumors. Inclusion will apply to children during active Physiotherapy, maintenance therapy or survivorship follow-up. Children who are receiving end-of-life or palliative care will not be included.

The study will exclude parents who have children currently in the intensive care unit or who have received cancer treatment in the last 24 to 48 hours or who have lost a child to death because these as such situations are possibly emotionally sensitive and may impact participants’ willingness to comfortable participate in phenomenological interviews.

### 2.5 Sampling strategy

The study will use criterion-based purposive sampling to recruit participants who satisfy the established inclusion standards. The study will permit eligible participants who include parents and primary caregivers of children aged 8 to 17 years with cancer who received physiotherapy rehabilitation after their diagnosis of leukemia lymphoma brain tumors or bone tumors or solid organ tumors. During the recruitment process, variation will be sought by including parents of children with different cancer types, different treatment phases, in-patient and out-patient experience, and across sociodemographic if at all possible.

The qualitative phenomenological research requires researchers to determine their sample size based on the depth and richness of their data rather than statistical power. Phenomenological studies focus on the depth, richness and meaningfulness of experiential accounts rather than the representativeness or statistical generalization. Previous methodological literature suggests that a sample size ranging from 6 to 12 participants may be sufficient to achieve data saturation in homogeneous groups (32, 33). The present study requires 10 to 15 participants to conduct a comprehensive investigation of parental experiences.

Participant recruitment will proceed until experiential and thematic saturation is reached, the point at which no new experiential meanings, themes or important findings will be obtained by a further interview. The achievement of saturation will be determined by consensus by the research team as data are being collected and analyzed through continued dialogue, and decisions will be recorded within the audit trail. Data collection will continue until thematic saturation is achieved, whereby no substantially new themes are identified during a predefined run length of interviews following an initial base size (A^+B^ approach where A is the Base size and +B is the Run length), indicating that the main thematic patterns have been sufficiently captured (32, 34). The researchers will determine the final sample size based on saturation, which will occur during data collection instead of using a predetermined numeric goal. In phenomenological health research, sample sizes are typically guided by depth of experiential data rather than statistical power, and studies commonly include small samples to allow for intensive, case-oriented analysis of lived experiences (35).

### 2.6 Semi-structured interview guide development

The research will utilize a semi-structured interview method to study how parents view physiotherapy rehabilitation for their children who undergo cancer treatment. The research team will create an interview guide which will help them conduct consistent interviews while achieving an in-depth examination of their subject matter. The initial version of the guide will contain twenty-five open-ended questions, which will enable researchers to study parents’ experiences and expectations about physiotherapy services. Interview questions will aim for an open narrative account of the parental lived experience, the meaning, emotions, perceptions of physiotherapy rehabilitation and will avoid leading or direct questioning. The use of neutral prompts and flexible probing questions will be employed to elicit meaningful phenomenological accounts from the participants. The research team will develop these questions through a comprehensive examination of current literature, earlier qualitative research and theoretical frameworks about Pediatric oncology rehabilitation and physiotherapy treatment.

The interview guide will be structured under two principal domains: (1) parents’ experiences with physiotherapy rehabilitation and (2) parents’ expectations regarding physiotherapy within pediatric oncology care. The open-ended format will allow participants to provide detailed narratives while enabling the interviewer to probe deeper into emerging themes. The semi-structured phenomenological interview technique is aimed at facilitating in-depth inquiry into the parents’ interpretation and meaning-making of their rehabilitation experience as part of their child’s cancer care. The research design allows qualitative research participants to express their views through their own words while researchers maintain study control over research objectives. Interviewer training will focus on experiencing how non-directive interviewing techniques and reflective listening can be effective in reducing interviewer influence, promoting talk, and creating opportunities for respondents to describe their lived experiences. The first draft of the semi-structured interview guide is tabulated in **Table 1**. The interview questions will be subsequently revised to prevent repetition in phrasing, ensure clarity, and promote phenomenological openness prior to proceeding to final data collection.

**Table 1:** Interview guide to collect parents’ experiences and expectations of children with cancer towards physiotherapy (version 1).

S no.	Questions
Experiences from Physiotherapy	
1	Could you tell me about your child’s health problem and treatment so far?
Probes	Chemotherapy, Radiation therapy, Surgery
2	When was physiotherapy first recommended for your child? Can you elaborate more on that?
Probes	Before, during, and after chemotherapy/radiation therapy/surgery
3	Can you describe your understanding and experiences regarding physiotherapy during your child’s cancer treatment
Probes	Exercises, daily activities, recovery
4	Has your child received physiotherapy? Please describe your experience in detail.
Probes	Physical experiences, emotional responses, challenges, memorable experiences
5	Could you describe your feelings when Physiotherapy was first introduced as a part of the treatment plan?
Probes	Emotional reactions, thoughts, concerns, expectations
6	Can you describe any experiences or changes you noticed after physiotherapy was introduced?
Probes	Improvement in daily activities
7	Could you explain how your child usually feels during and after physiotherapy sessions?
Probes	Physical experiences, emotional experiences, comfort, discomfort
8	What kind of activities or exercises does your child do in physiotherapy?
Probes	Types of rehabilitation activities, participation experiences, comfort with activities
9	Could you explain the challenges you, as a parent, faced during the Physiotherapy treatment?
Probes	practical difficulties, emotional concerns, financial or logistical challenges
10	Can you describe any emotional or behavioral experiences you observed in your child during physiotherapy rehabilitation?
Probes	Calm, agitated, reluctant
11	Could you explain the difficulties you have in attending the physiotherapy sessions regularly?
Probes	Practical barriers, travel-related experiences, child-related difficulties
12	Can you describe your experiences with the information or guidance you received regarding physiotherapy rehabilitation?
Probes	Exercise tips, home exercise advice
Expectations from Physiotherapy	
13	Can you describe your perceptions regarding the role of physiotherapy during your child’s cancer treatment?
Probes	Improves strength and activities of daily living, helps the child move better
14	Can you describe how comfortable you are in bringing your child to physiotherapy sessions?
Probes	Comfort, difficulties, practical experiences
15	Can you describe your experiences regarding how physiotherapy may have influenced your child’s daily life and overall well-being?
Probes	Sitting, standing, walking/playing, overall Independence
16	What are your thoughts and experiences regarding physiotherapy as part of your child’s recovery process?
Probes	walking, sitting, playing, self-care
17	Could you explain the importance of physiotherapy in your child’s overall recovery?
Probes	Expectations, concerns, rehabilitation goals, hopes regarding recovery
18	Can you share your expectations for your child’s rehabilitation through Physiotherapy?
Probes	Pain relief, improve activities of daily living, child fear, child enjoy
19	What are your thoughts regarding continuation of physiotherapy rehabilitation over time?
Probes	Benefit for the child, recovery, and strength
20	Can you describe whether physiotherapy rehabilitation aligned with your expectations and experiences regarding your child’s care?
Probes	Getting better, Positive attitudes
21	Please explain the experiences of support that you receive from the family during the Physiotherapy treatment session.
Probes	emotional support, encouragement
22	Please explain the experiences of support that you receive from the healthcare staff during the Physiotherapy treatment session.
Probes	Guidance/help, communication
23	Would you elaborate on what kind of support helps you to continue physiotherapy for your child?
Probes	Government scheme, Insurance, workplace support, family support
24	Can you please explain how comfortable you feel when discussing your concerns with the physiotherapist?
Probes	Comfort, communication experiences, concerns
25	Can you describe any suggestions or changes you feel may improve physiotherapy rehabilitation services for children with cancer?
Probes	Functional, day-to-day life-related activities, school-based activities

The interview guide will be tested for methodological rigor through a content validation procedure which employs the Item-level Content Validity Index and the Scale-level Content Validity Index (13). The I-CVI evaluates the relevance of individual items in the instrument. Experts will independently assess each question for its clarity, relevance, and appropriateness to the research objectives. Each item will be rated using a four-point ordinal scale for relevance and clarity during content validation. The I-CVI for each item will be calculated as the proportion of experts who rate the item as relevant. According to established methodological guidelines, an I-CVI value of 0.78 or higher is considered acceptable when evaluated by panels consisting of six or more experts, indicating that the item adequately represents the intended construct.

The S-CVI assessment will evaluate the complete content validity of the interview guide through its individual item validation assessment. The S- CVI was calculated using two methods: the universal agreement approach (S- CVI/UA) and the averaging approach (S- CVI/Ave), which represents the average of the I-CVI scores across all items in the instrument. Scale-level content validity will reach excellent status when its results show a threshold value that equals 0.90 or higher. The indices together show that the interview guide provides proof of both individual item importance and complete content area measurement.

A panel of 8–12 subject experts, including pediatric physiotherapists, pediatric oncologists and qualitative research experts, together with academicians who have worked in pediatric rehabilitation, will assess the draft interview guide. Expert opinion will be based on clinical, academic or research expertise in the areas of rehabilitation of children with cancer, qualitative research and children’s physiotherapy. The content validation process will use a structured online Delphi method, which enables experts to assess content while keeping their identities private. This method decreases the power of strong viewpoints to shape results while it provides experts with a system that helps them reach agreement.

Multiple rounds of evaluation with the same group of experts will be undertaken using the Delphi method to gain expert agreement on the clarity, appropriateness and relevance of the interview questions. Consensus was reached when the agreement among experts was 80% or more. Experts will assess each item for clarity, relevance, and alignment with the study objectives. After receiving responses from the required number of experts, the survey link will be closed, and the responses will be analyzed by the researchers. The expert feedback will result in item changes which include rephrasing, merging items, or removing items to improve clarity, conceptual relevance and contextual suitability. Expert’s qualitative comments and recommendations will also be taken into consideration and included where relevant. These comments would help enhance the conceptual clarity and contextual situation of the interview guide. (36–39) The research team will conduct multiple evaluation rounds using new expert panels to assess the updated interview guide until they achieve the necessary content validity requirements.

The research team will establish the final validated interview guide after they complete the Delphi rounds and achieve acceptable I-CVI and S-CVI values. The validated guide will function as the main data collection tool for semi-structured interviews with parents whose children receive physiotherapy rehabilitation at pediatric oncology facilities.

### 2.7 Semi-structured interview guide administration

The finalized semi-structured interview guide will be used to conduct in-depth interviews with eligible participants. The hospital will use face-to-face interviews, which require a quiet, well-lit and well-ventilated room for their interview process because this setup protects privacy while reducing interruptions. The interview will be recorded using the Sony ICD- PX470 Digital Voice Recorder. The interview process will use telephone interviews when face-to-face interviews cannot take place, because the interviewers will create a proper setup for a clear audio recording to occur. The interview sessions will only include the principal investigator and the participant because this setup protects participant privacy and promotes free dialogue. Interviewers who will help in collecting data will have sufficient training to conduct qualitative interviews and will have knowledge of the Kannada and English languages so that they can communicate well with participants and perform a phenomenological interview. All interviews will be undertaken by trained research personnel independent of direct clinical involvement with the children being studied to limit potential power imbalance and response bias. Phenomenological interviewing will be used during the interviews to facilitate participants’ description and reflection on the experiences, meaning, and emotions of the physiotherapy rehabilitation during their child’s cancer treatment experience.

The principal investigator (D.K) conducts the interviews and is a qualified physiotherapist experienced in pediatric oncology rehabilitation and has trained in qualitative research. The study’s co-investigator (A.J.S) is an experienced qualitative researcher. He will advise on interviews, including training the principal investigator and offering supervision to maintain rigor in qualitative research. The participants will have no established prior personal relationship with the principal investigator. The participants will be selected by the treating clinical team, and informed consent will be obtained in writing. Participant consent will be obtained before each interview. The principal investigator of this study will take reflexive notes throughout to account for any possible bias.

The interview guide will include open-ended questions, which will help researchers understand how parents experience the rehabilitation process and what they expect from physiotherapy treatment for their child during cancer therapy. The research will investigate areas which include understanding physiotherapy services, perceived benefits, rehabilitation difficulties, communication and practical healthcare experiences and future treatment expectations.

The research team will conduct audio recordings of interviews only after participants provide their written consent. The researchers will use field notes to capture all non-verbal communication and emotional displays, and the various contextual elements of the study. Reflexive notes will also be recorded throughout the interview process to account for interviewer reflections, contextual observations, and impacts on data collection. The research team will create audio recordings, which they will transcribe word for word and eliminate all personal identification information before storing the content on secure devices that only authorized members can access. The interviews recorded in the Kannada language will initially be verbatim transcribed in the original language and then translated in English for analysis. The accuracy of translation and preservation of the meaning in context will be scrutinized by researchers who know both English and Kannada well. Discrepancies will be negotiated by the research team members to ensure cultural and conceptual integrity (40). The research team will analyze data through thematic analysis, which follows the six-phase framework developed by Braun and Clarke to process transcripts into coded systems that reveal common patterns and meanings and themes associated with parents’ lived experiences, meanings, perceptions, and rehabilitation experiences.

### 2.8 Data analysis

The data analysis process will begin as soon as the researchers finish conducting their initial interview. The research team will create word-for-word transcriptions of all interviews, which two researchers will examine separately to maintain research quality standards. The team will start by establishing meaning units, which lead to the first code development and which later support the creation of an initial coding system. The primary investigator will conduct the research analysis, while the secondary investigator will participate in ongoing meetings to check coding accuracy, settle differences and develop new categories through consensus building. Reflexivity will also take place throughout the analysis as a critique on the researchers’ emphases’ and assumptions and how it may affect the formulation of themes. The team will develop an audit trail of all analysis work. Methodological transparency will be increased by maintain records of analytic decisions in the phenomenological analysis.

Thematic analysis will be conducted following Braun and Clarke’s six-phase framework (33, 41). Reflexive thematic analysis was deemed suitable for the current phenomenological research as it is concerned with exploring conceptually-driven themes across the ideological world of participants’ lived experiences while also acknowledging interpretative and subjective variability (33). While thematic analysis may not be phenomenological per se, its reflexive and experiential emphasis makes it appropriate for phenomenological studies with investigations into participants’ subjective meaning and interpretations.

Experiences of physiotherapy rehabilitation, how the participants represent, conceptualize and make meaning of their individual experiences of physiotherapy rehabilitation are the focus of this discussion. Themes will thus emerge from shared meanings within participants’ representations, and not from predefined categories of parental attitudes or confounding factors.

The first phase requires researchers to read the transcripts multiple times while they study the material. The second phase will begin with the creation of fundamental codes which the researchers will apply to the entire dataset through an inductive process which allows themes and meanings to emerge inductively from participants’ lived experiences rather than being imposed through predetermined theoretical assumptions. The third phase will involve analyzing codes to find patterns and similarities and develop relationships between different concepts which will lead to the creation of potential themes. Special emphasis will be placed on highlighting experiential meanings, emotional reactions, caregiving experiences and contextual meanings of physiotherapy rehabilitation in pediatric oncology practice. The fourth stage requires an evaluation process to examine the themes which will use both coded extracts and complete dataset to determine whether themes maintain internal consistency and unique characteristics. The fifth phase will define and name themes through an explanation of their research scope and content and their importance to research goals. The sixth and final phase involves organizing and reporting the findings, incorporating illustrative participant quotations to illustrate lived experiences, contextual meanings, and phenomenological interpretations generated from the data.

The researchers will use MAXQDA qualitative data analysis (42) software to create structured coding systems, which will help them organize and access their research data. The research will achieve its goals through three methods: independent review and reflexive discussions, the establishment of an audit trail, which will enhance the credibility, dependability and confirmability of the research findings. Peer debriefing and continuing reflexive discussion among members of the research team will be undertaken to bolster the credibility of analysis and consistency of interpretation.

### 2.9 Rigor and trustworthiness

The qualitative research study will maintain methodological integrity by applying established trustworthiness standards, which include credibility, dependability, confirmability and transferability. For the purpose of the context and transferability of findings, a detailed account of the characteristics of the participants and treatment settings, and information about the rehabilitation context will be given.

#### 2.9.1 Triangulation

The researchers will achieve data triangulation through their use of multiple sources that include both in-depth interviews and field notes. Contextual observation and field notes will be used to further interpret the lived experiences of the participants and understand the context in the data analysis. The research team will maintain investigator triangulation by including multiple researchers who will work together to code and analyze data which will enable them to compare and improve their findings.

#### 2.9.2 Independent coding

The principal investigator (D.K) will conduct independent coding of all interview transcripts through the efforts of two researchers to enhance reflexive discussion, interpretive depth, and analytic transparency. The researchers will create initial codes through inductive development from the collected data and the codes will be verified by another researcher (A.J.S) who is not part of active data collection. Through comparison between different researchers. The team will discuss and reach agreement on coding discrepancies to maintain consistent results while reducing personal bias in their understanding of the material.

#### 2.9.3 Audit trail

The research process will maintain an extensive audit trail, which will document all research activities. The research process will document all research activities through the creation of three specific documents, which will document methodological decisions, reflexive observations, coding development, and interpretive processes throughout the study. The research process will be transparently documented through detailed records which enable outside parties to assess the research activities.

#### 2.9.4 Reflexivity

Reflexivity will be preserved through a better understanding of the researcher’s backgrounds and their established assumptions, and their potential effects on data analysis results. The reflexive stance involves a continual critical analysis of the researchers’ assumptions, professional background, researcher-participant interaction, and researcher as the interpreter of data collection and analysis. The researchers will engage in continuous self-reflection and they will maintain reflexive notes throughout the study in order to assess their influence on data collection and analysis methods. The researchers will also need to remain mindful of how their physical therapy and rehabilitation backgrounds may influence their understanding of participants’ experiences and meanings. The research team will conduct regular discussions to support reflexive practice while ensuring that findings stay connected to participants’ viewpoints. Throughout the study the research team will hold regular peer debriefing discussions to increase the interpretive credibility and analytical consistency of the findings.

## 3 Results

As this is a study protocol, there are no results obtained. Data collection has not yet been completed at the time of manuscript submission. The research study design received no input from patients or members of the public, but researchers will use the early data collection phase to assess interview guide testing and preliminary discussions, which were tested during the pilot phase. The researchers will conduct thematic analysis to analyze transcribed verbatim interviews, which will produce codes, subthemes and main themes. The research team will analyze data without considering participants’ gender or socioeconomic status or their children’s diagnosis to obtain different perspectives from parents. The research is expected to provide qualitative understandings of the parents’ experiences, expectations, perceptions and difficulties of physiotherapy rehabilitation for children with cancer. The expected outcomes could have implications for refining family-centered rehabilitation approaches and enhancing the integration of physiotherapy within pediatric oncology care pathways.

## 4 Discussion

The research protocol investigates an important unexplored area of Pediatric oncology rehabilitation. Parents of children with cancer in low- and middle-income countries need more research to understand their views about how physiotherapy and structured rehabilitation programs should become part of their child’s cancer treatment. By focusing on parents’ lived experiences and meaning-making processes, the study seeks to deepen understanding of how physiotherapy rehabilitation is interpreted, experienced, and integrated into family caregiving realities during pediatric oncology treatment (43). Parents who serve as primary caregivers and decision-makers for their child’s cancer treatment path need rehabilitation programs which match their personal beliefs and expectations and life experiences. The research uses descriptive methods to create a detailed localized understanding of how Indian parents understand and use physiotherapy rehabilitation during their child’s cancer treatment. Rehabilitation models need this information to create solutions which respect cultural differences and assist families, while physiotherapy needs this data to build effective pathways for pediatric oncology care.

The study establishes various methodological strengths which provide both increased research accuracy and authentic research findings. The qualitative framework enables researchers to study how parents create their understanding of medical procedures that take place in a healthcare setting which operates under specific cultural norms. The interview guide development process required structured Delphi method implementation together with Content Validity Index (CVI) evaluation which enhanced the instrument’s content validity and contextual applicability. The research team will be applying Braun and Clarke’s six-phase process together with qualitative data analysis software to execute their inductive thematic analysis framework which enabled them to perform their coding work and create their research themes in an understandable way. Reflexive practices, bracketing process, and continuous interpretive dialogues between researchers also serve to increase phenomenological rigor and transparency during analysis of participants’ lived experiences. The research methods which include independent evaluation and reflexive discussions together with audit trail management, provide evidence of research findings which have high credibility and dependability with all information being easily verified. Research methods need to reach high levels of reliability because they create qualitative evidence which medical professionals and service designers and rehabilitation policy makers need for their work.

The expected results will have significant consequences for both medical practice and policy development. The research identifies parental factors that enable or hinder their support of physiotherapy rehab which will lead to better parent training programs and better collaboration between oncologists and physiotherapists who work together to make medical decisions. Further by exploring parents’ feelings, problems during caregiving and expectation of rehabilitation could provide guidance to the development of culturally sensitive and family centered physiotherapy rehabilitation services in pediatric oncology ward. The institutional standards which develop from actual human experiences will create rehabilitation programs which become standard practice in oncology treatment instead of being available as additional voluntary services. The study will help advance advocacy efforts which aim to establish pediatric oncology rehabilitation as a fundamental part of national survivorship programs in India.

The research outcomes of this study will be limited to the particular setting of one tertiary care hospital which will affect their applicability to different medical facilities and cultural contexts. In addition, phenomenological findings are inherently context-dependent and interpretive in nature, which may limit broader transferability beyond similar healthcare and sociocultural settings. However, it is expected that the participants represent different geographical regions, because of which, the study findings can have broader relevance and be applicable across various contexts. Even though the proposed sample size is minimum 15 participants, the sample will be verified with data saturation and thematic saturation. Moreover, because of their context-specific and interpretive character, phenomenological data have restricted transferability to similar health and social environments.

The upcoming research will investigate the relationships between parental views and rehabilitation success through multi-center studies and long-term research and mixed-methods research that examines the link between parental views and functional achievement. The predicted results will likely further strengthen the evidence for the incorporation of structured physiotherapy rehabilitation into pediatric oncology care in low- and middle-income settings.

## 5 Conclusion

This qualitative, phenomenological study aims to investigate the experiences and meaning-making of Indian parents in their context of their child’s cancer rehabilitation. By gaining understanding of how parents conceptualize and make meaning of rehabilitation from their caregiver and cultural perspective, it is hoped that this will contribute information useful for a contextually relevant, family-centered and culturally-sensitive rehabilitation practice in pediatric oncology.
